# High-Throughput Screening to Identify Plant Derived Human LDH-A Inhibitors

**DOI:** 10.9734/ejmp/2013/5995

**Published:** 2013

**Authors:** S. Deiab, E. Mazzio, S. Messeha, N. Mack, K. F. A. Soliman

**Affiliations:** 1 College of Pharmacy and Pharmaceutical Sciences, Florida A & M University, Tallahassee, Florida 32307, USA

**Keywords:** Lactic acid, Warburg, LDH-A, inhibitor, herbs, medicinal plants

## Abstract

**Aims:**

Lactate dehydrogenase (LDH)-A is highly expressed in diverse human malignant tumors, parallel to aggressive metastatic disease, resistance to radiation/chemotherapy and clinically poor outcome. Although this enzyme constitutes a plausible target in treatment of advanced cancer, there are few known LDH-A inhibitors.

**Study Design:**

In this work, we utilized a high-throughput enzyme micro-array format to screen and evaluate > 900 commonly used medicinal plant extracts (0.00001-.5 *mg/ml*) for capacity to inhibit activity of recombinant full length human LDHA; EC .*1.1.1.27*.

**Methodology:**

The protein sequence of purified enzyme was confirmed using 1D gel electrophoresis- MALDI*-TOF-*MS/MS, enzyme activity was validated by oxidation of NADH (500μM) and kinetic inhibition established in the presence of a known inhibitor (Oxalic Acid).

**Results:**

Of the natural extracts tested, the lowest IC_50_s [<0.001 mg/ml] were obtained by: Chinese Gallnut (*Melaphis chinensis gallnut)*, Bladderwrack (*Fucus vesiculosus)*, Kelp (*Laminaria Japonica*) and Babul (*Acacia Arabica)*. Forty-six additional herbs contained significant LDH-A inhibitory properties with IC_50_s [<0.07 mg/ml], some of which have common names of Arjun, Pipsissewa, Cinnamon, Pink Rose Buds/Petals, Wintergreen, Cat’s Claw, Witch Hazel Root and Rhodiola Root.

**Conclusion:**

These findings reflect relative potency by rank of commonly used herbs and plants that contain human LDH-A inhibitory properties. Future research will be required to isolate chemical constituents within these plants responsible for LDH-A inhibition and investigate potential therapeutic application.

## 1. INTRODUCTION

Human lactate dehydrogenase is a tetrameric enzyme [1] highly expressed in smooth muscle. The LDH subtype A is up-regulated in diverse tumor tissues [2] including lung [3], pheochromocytoma, paraganglioma [4], esophageal squamous cell carcinoma [5], breast [6], endometrial adenocarcinoma, ovarian cystadenocarcinoma [7], hereditary leiomyomatosis renal carcinoma [8] and colon carcinoma [9–11]. Unlike normal differentiated cells where lactate accumulation occurs anaerobically, cancer cells readily convert glucose into lactate aerobically, a phenomenon termed the Warburg effect [12]. Elevated protein expression or enzyme function of LDH–A is a contributor to not only accumulated lactate, but also aggressive tumor growth [13], advanced progression [14], metastasis [15–17], acidity [18], and subsequent resistance to radiation and chemotherapy [19–22]. LDH–A knockdown, or lowering the functional capacity of LDH-A can suppress tumor growth and metastasis [23], indicating that this enzyme could serve as a novel targeted cancer therapy strategy. In this study, we elucidate LDH–A inhibitory effects of commonly used medicinal herbal extracts rom around the world.

## 2. METHODOLOGY

Hanks Balanced Salt Solution, (4-(2-hydroxyethyl)-1-piperazineethanesulfonic acid) (HEPES), iodoacetamide, DL-Dithiothreitol (DTT), ethanol, 96 well plates, general reagents and supplies were all purchased from Sigma Scientific (Sigma, St Louis MO), LDH-A was purchased from Abcam (Cambridge, MA). Natural products were provided by Frontier Natural Products Co-op (Norway, IA), Montery Bay Spice Company (Watsonville, CA), Mountain Rose Herbs (Eugene, OR), Mayway Traditional Chinese Herbs (Oakland, California), Kalyx Natural Marketplace (Camden, NY), Futureceuticals (Momence, IL), organic fruit vegetable markets and Florida Food Products Inc. (Eustis, FL).

### 2.1 Herbal Extraction

Plant and herbal extracts were macerated, diced, chopped and homogenized in 100% ethanol at 50mg/ml. Samples were placed on a rocker shaker for 24 hours and stored in air tight containers at −20°C in the dark. All serial dilutions were made using a diluent consisting of HBSS with 10mM HEPES adjusted to a pH 7.4.

### 2.2 MALDI MS/MS Protein Identification

Recombinant full length Human LDHA (amino acids 1–332) with N terminal His tag; 352 amino acids with tag, MW 38.8 kDa: Enzyme Commission (EC) Number 1.1.1.27 (BRENDA | IUBMB) (Abcam, Cambridge, MA) was utilized. The protein was validated by proteomic analysis using Matrix Assisted Laser Desorption Ionization (MALDI) Mass Spec (MS/MS) and analyzed by Mascot ID. Briefly, pure enzyme was solubilized, denatured and subjected to 1 D SDS page gel electrophoresis using a 5–20% Tris-HCL gradient gel with a running buffer 25 mM Tris, 192 mM glycine, 0.1% SDS at 200 V for 35 minutes. High intensity bands for LDH-A at 38 KD were visualized with G-Biosciences’ LabSafe GEL Blue^™^ stain, then excised, followed by in gel digestion of peptides with trypsin, followed by reduction/alkylation with DTT and iodoacetamide, respectively. Samples were analyzed using MALDI MS/MS (Applied Biosystems) and protein sequence identified by Mascot analysis.

### 2.3 LDH-A Activity

A continuous LDH-A assay was used to conduct high-throughput screening (HTS). Briefly, a buffer consisted of HBSS + calcium and magnesium pH adjusted to 7.0. LDH-A enzyme (final concentration .02 Units/ml) was added to treatments of tier one, with concentrations of .5 mg/ml. After addition of β-Nicotinamide Adenine Dinucleotide, Reduced Form Solution (β-NADH) (final working concentration of 500μM) a pre-reading @ 340nm was established and the reaction was started with a solution of substrate pyruvate (final concentration = 3mM).

### 2.4 High Throughput Design

A rapid screening model was used based on works previously described [24]. An enzyme micro-array format was adapted to where a 96 well plate contained a known concentration of enzyme, and treatments of equal concentration dissolved in buffered HBSS and β-NADH. After addition of the substrate (pyruvate) a curve for time dependent NADH oxidation was monitored continuously over 75 minutes @340nm. A first tier investigation was established at a final working concentration of 0.5 mg/ml for each herbal extract. All compounds that inhibited LDH-A with in the first tier screen below 50% of control, were then placed in a second tier (final concentration = 0.25 mg/ml), third tier (final concentration =0.1 mg/ml) and fourth tier (final concentrations with extended range at 0.006, 0.03 and 0.16 mg/ml). Extracts were ranked for potency, and the most potent were further evaluated over a minimum of 6 concentrations from 1mg/ml to less than 0.00001 mg/ml to establish an IC_50_. The enzyme micro-array format was rapid, reproducible and repeatedly corroborated by a four-tier evaluation process.

### 2.5 Data Analysis

Statistical analysis was performed using Graph Pad Prism (version 3.0; Graph Pad Software Inc. San Diego, CA, USA) with significance of difference between the groups assessed using a one-way ANOVA, followed by Tukey post hoc means comparison test, a two way ANOVA or Student’s t test. IC_50_s were determined by regression analysis using Origin Software (OriginLab, Northampton, MA).

## 3. RESULTS AND DISCUSSION

**Fig. 1:**
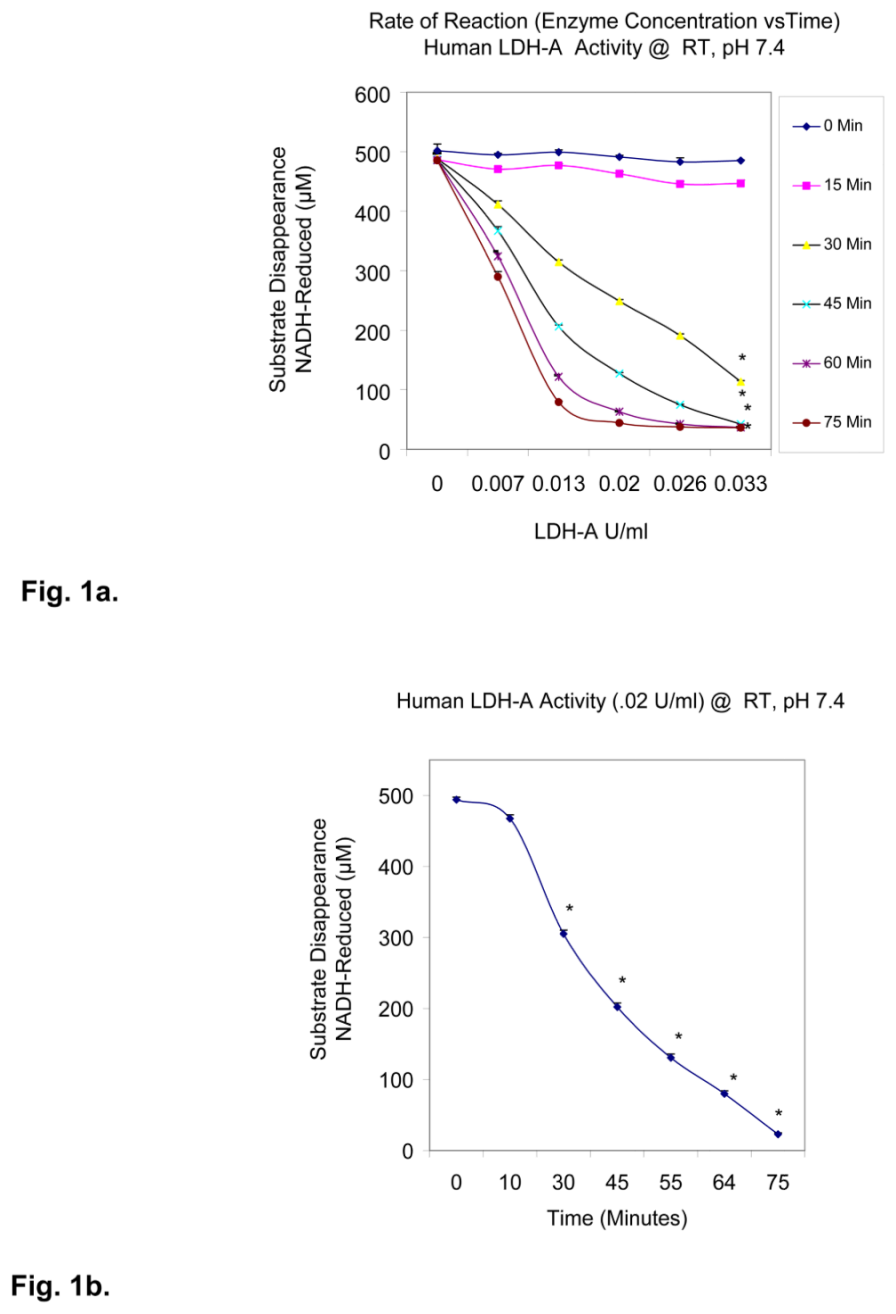

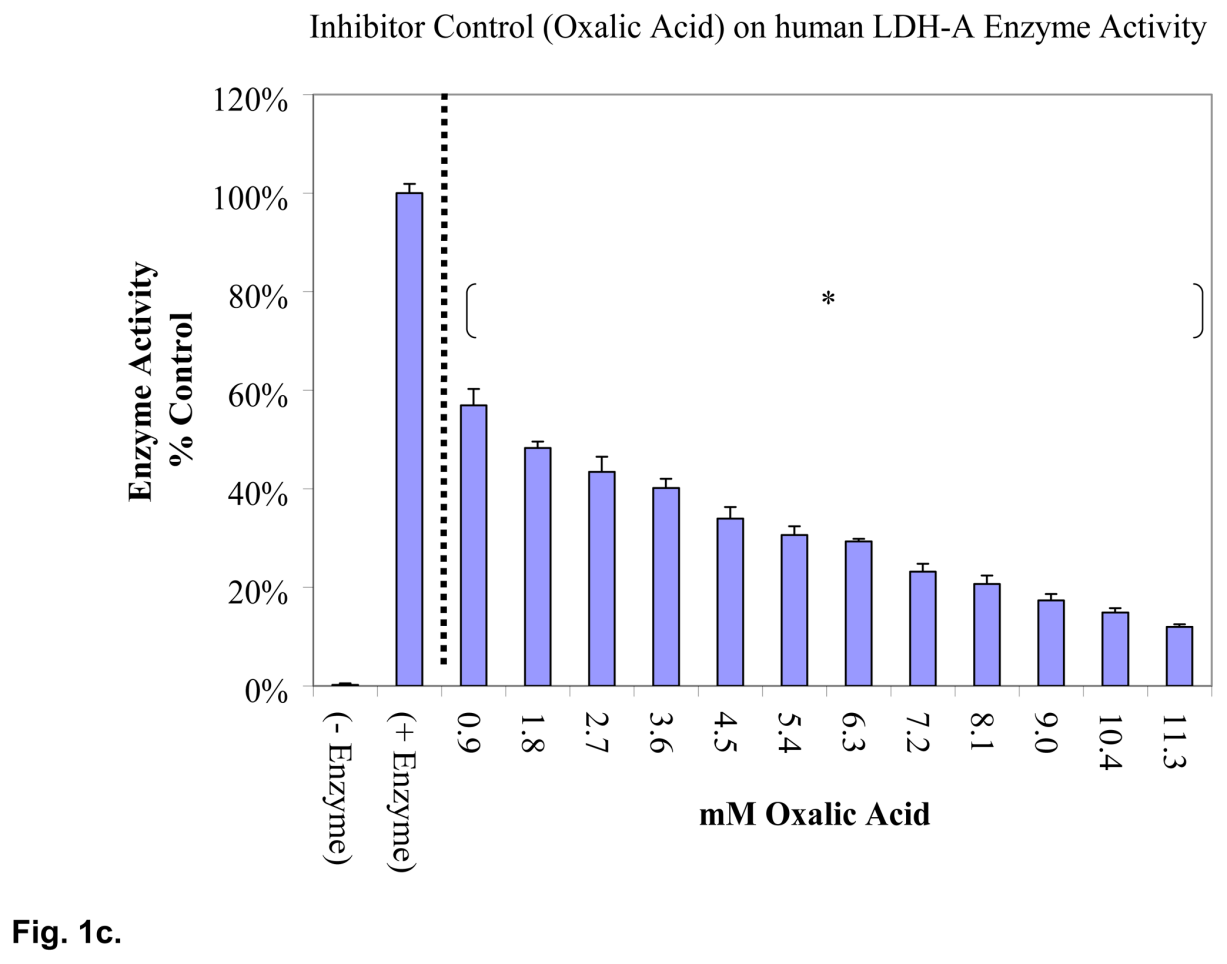
Fig. 1a. Human LDH-A Activity-time and enzyme concentration dependent NADH oxidation in the presence of 3mM pyruvate. The data represent μM NADH reduced from 0–75 minutes (incubation at RT) and are presented as the Mean ± S.E.M, n=4. Significance of difference for product formation between Time _0_ vs Time _15 – 75 minutes_ were determined using a two-way ANOVA.* p< 0.05 Fig. 1b. *Human* LDH Activity at .02 Units/ml with time dependent NADH oxidation in the presence of 3mM pyurvate. The data represent μM NADH and are presented as the Mean ± S.E.M, n=4. Significance of difference for product formation between Time _0_ vs Time _75 minutes_ was determined using a one-way ANOVA followed by a Tukey post hoc test. * p<0 .05 Fig. 1c. Human LDH Activity – Inhibitor Control. The data represent % Enzyme Activity @ 75 Minutes and are presented as the Mean ± S.E.M, n=4. Significance of difference for enzyme activity between the control and oxalic acid (0.9–11.3 mM) was determined using a one-way ANOVA followed by a Tukey post hoc test. * p<0 .05

Method validation was established by monitoring continuous NADH oxidation initiated by addition of the substrate pyruvic acid (3mM) in the presence of varying enzyme concentration/time (Fig. 1a)

A screening validation process was established using 0.02 U/ml LDH-A over 75 minutes (Fig. 1b) ± a known inhibitor; oxalic acid (Fig. 1c). The data shows a slow but steady rate of reaction, resulting in time dependent O.D. decay over 65–70 minutes with high signal/noise ratio.

**Fig. 2:**
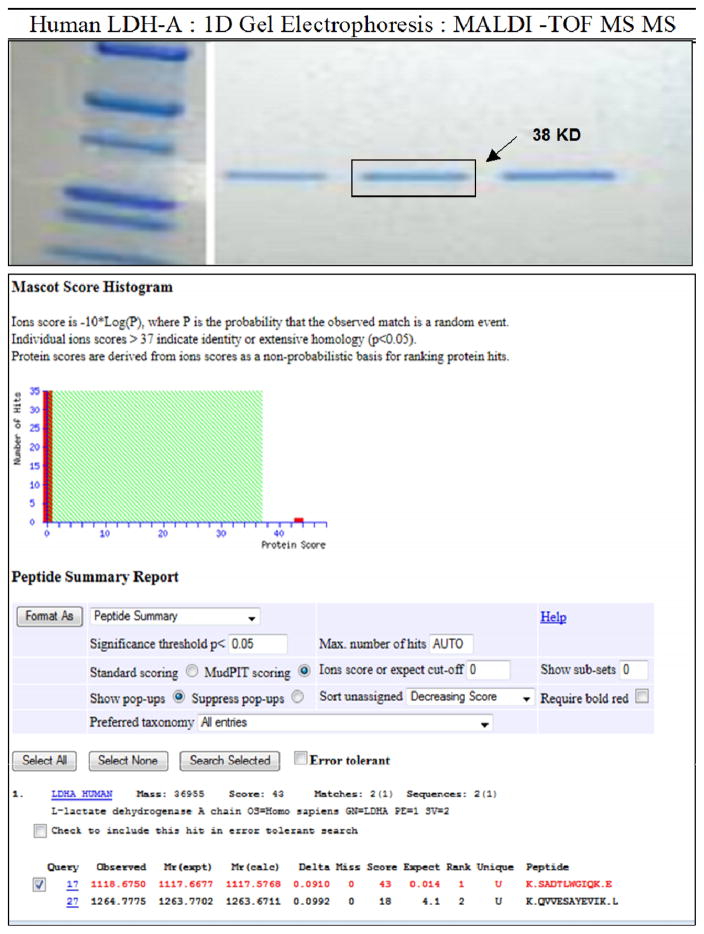
Mascot results for protein identification by peptide mass fingerprinting of Human LDH-A tryptic digest analyzed by MALDI-TOF/TOF-MS

Although the enzyme used in this screening, was described as a recombinant full length Human LDHA (amino acids 1–332) with N terminal His tag; 352 amino acids with tag, MW 38.8 kDa: Enzyme Commission (EC) Number 1.1.1.27 (BRENDA | IUBMB) (Abcam, Cambridge, MA), we confirmed the identity of the enzyme using Matrix Assisted Laser Desorption Ionisation (MALDI) Mass Spec (MS/MS) and analysis by Mascot ID (Fig. 2). Fig. 2 (Top panel) shows the 1 D SDS page gel electrophoresis of the purified enzyme at three concentrations (right), along with a molecular marker standard (left). The gel band was excised, digested and analyzed by MSMS for peptide sequence and protein identify (Bottom Panel). The data showed a positive hit for human LDH-A with a 95% confidence interval for peptide/sequence mass.

**Fig. 3:**
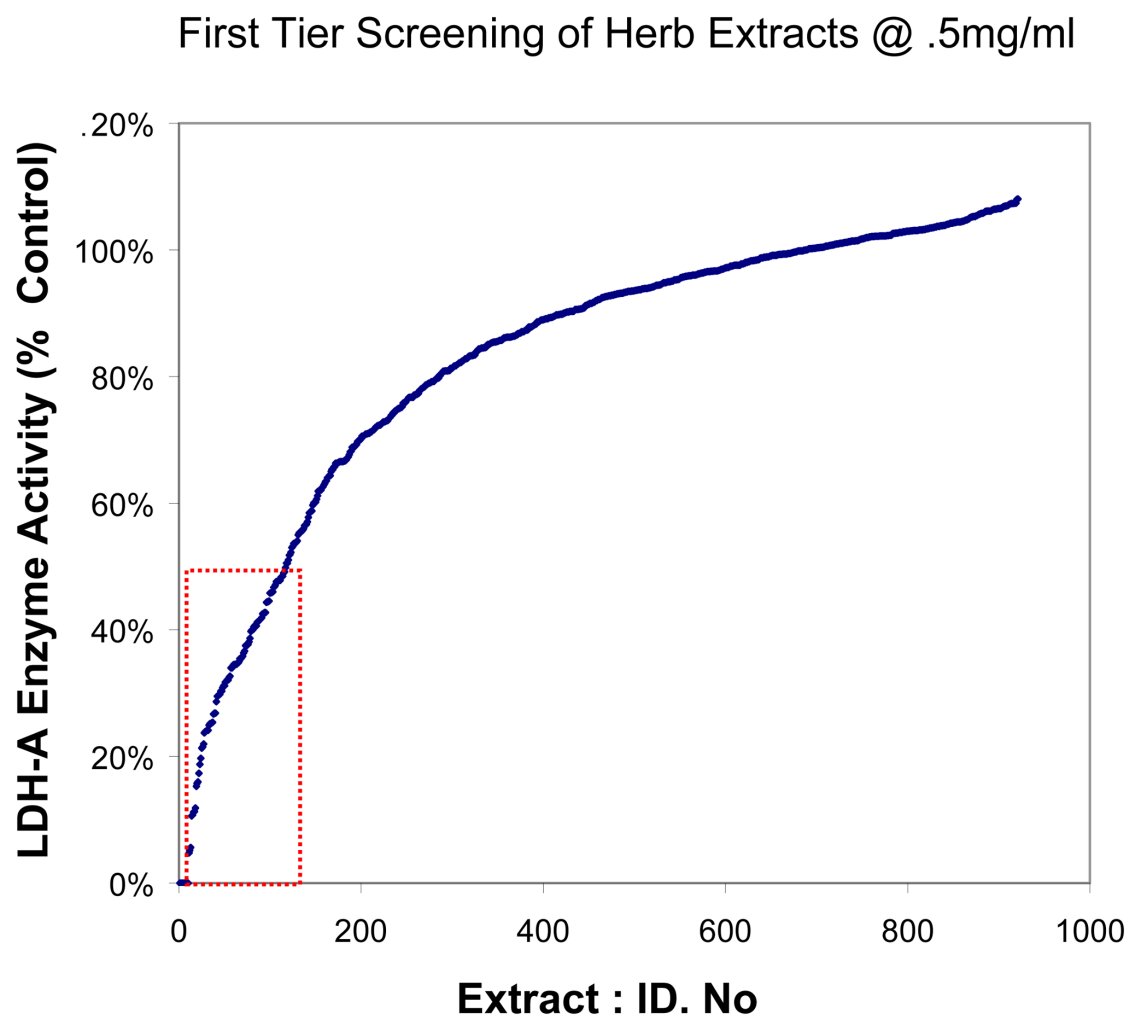
A high-throughput enzyme experimental micro-array design. 905 extracts were evaluated for capacity to inhibit *Human* LDH-A. A first tier screening was conducted at a final working concentration of 0.5 mg/ml for each herbal extract. Enzyme activity was continuously monitored over a 75 min period. Extracts demonstrating an IC_50_ <0.5 mg/ml (red dotted line -----) were screened through subsequent tier evaluations

A high throughput enzyme micro-array model was used in this work. Over 900 extracts of equal concentration (0.5 mg/ml) were dissolved in buffered HBSS and incubated with the enzyme, for 5 minutes prior to start of the reaction. After addition of the substrate, a curve for time dependent product formation was monitored continuously over 75 minutes. Fig. 3 represents the 75 minute reading taken from the original screening with values for each compound sorted according to inhibitory potency. Of the initially tested extracts, only 115 inhibited LDH-A within the first tier below 50% of control, denoted by red dotted line --- (Fig. 3). These plant extracts were then subject to a second tier screenings (final concentration = 0.25 mg/ml), third tier screenings (final concentration =0.1 mg/ml) and fourth tier screenings (final concentrations with extended range at 0.006 to 0.16 mg/ml) to which regression analysis was used to calculate IC_50_s.

**Fig. 4:**
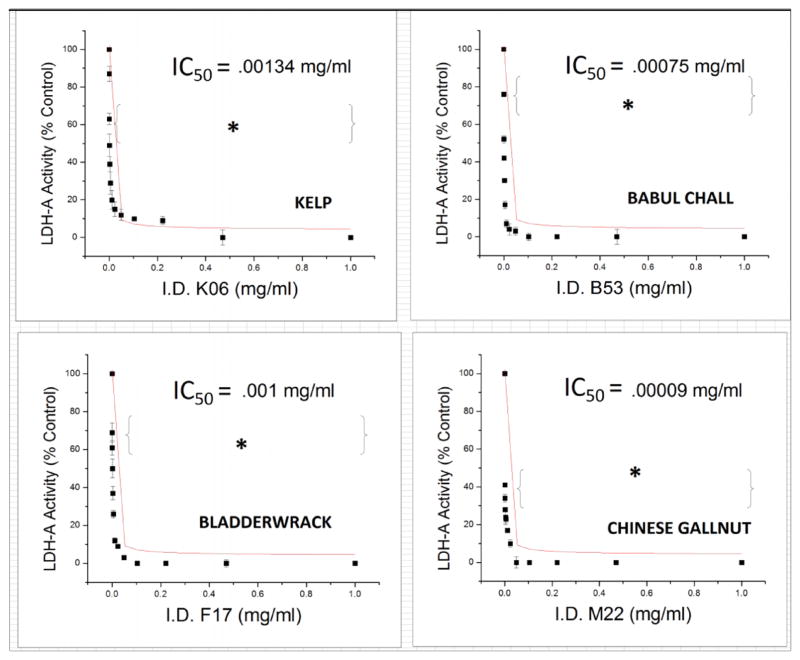
Most Potent Herbal Extract Inhibitors of *Human* LDH-A activity. The data represent LDH-A activity as % control in the presence or absence of extracts and are presented as Mean ± S.E.M., n=4. IC_50_ concentrations were established from a sigmoidal fit dose-response equation and significance of difference between the controls vs. treatment was determined using a one-way ANOVA followed by a Tukey post hoc test. * p<0.05

Of the 115 retested, 46 extracts showed an IC_50_ <0.077 mg/ml Table 1 as listed by potency rank. Full inhibitory dose response curves are shown for the top four inhibitors (Fig. 4).

Table 1 Human LDH-A inhibitors by potency. Extract IC_50_s are listed by both mg/ml and μg/ml for inhibiting NADH oxidation on .02U/ml of LDHA.

## 4. DISCUSSION

In this HTS study, we investigated the ethanol extract of 905 natural products to identify those with human LDH-A inhibitor properties. Our results show Melaphis chinensis gallnut, also known as Rhus chinensis (RC) nut, to be the most potent and within a therapeutic range. Rhus chinensis belongs to family Anacardiaceae and genus Rhus. This family, consists of 250 species found in China [25] and many other locations around the world. Numerous traditional Chinese herbalists recommend RC for ailments such as chronic cough due to lung deficiency, chronic diarrhea and for clearing toxins. Unfortunately, these claims are not based on scientific grounds, yet the interest in this herb continues to increase for its numerous scientifically based findings. There is an abundance of research on the biological and pharmacological benefits of RC. For example, it was shown to be very effective in harmful intestinal and periodontal bacterial growth inhibition by a mechanism mediated in parts by its constituent’s gallic acid and gallotaninns [26–28]. As an antiviral, RC ethyl acetate extract has inhibitory effects against hepatitis carcinoma virus [29,30] and severe acute respiratory syndrome corona virus [30]. Penta-1,2,3,4,6-O-galloyl-β-D-glucose (PGG) isolated from RC shows promising hepatoprotective properties [31] and the anticancer effects of RC are believed to involve inhibition of dcd25A phosphotase activity [32] or gallic acid as one of the bioactive components in RC [26,33,34] which directly induces apoptosis in prostate cancer cells [35]. PGG was also shown by Huh et al, to be a constituent in RC with ability to inhibit angiogenesis and stimulate apoptosis [36]. In addition, PGG reduced cancer cell viability [37] as well as suppressed prostate cancer, bone metastasis [38] and caused cell cycle arrest at the G1 phase [39].

## 5. CONCLUSION

The findings in this study, while broad show that a number of natural products have the ability to inhibit LDH-A, which may adversely affect cancer cell survival. We present evidence for LDH-A inhibitory properties of a number of commonly used herbs and spices with previously reported anti-cancer properties including bladderwrack [40], kelp [41], cinnamon [42], cats claw bark [43], arjun [44], polygonum multiflorum [45] and witch hazel [46]. Future research will be required to evaluate if LDH-A inhibition is a contributing factor to tumoricidal or anti-proliferative properties of these herbs on diverse human cancer cells.

## Figures and Tables

**Table 1:** Natural source aqueous extracts with human LDH-A inhibitory potency by rank

Rank	ID No.	Common ± Chinese Name	*Scientific Name*	IC 50 mg/ml	IC 50 μg/ml
1	M22	Wu Bei Zi	*Melaphis chinensis gallnut*	0.00009	0.09
4	B53	Babul Chall Bark	*Acacia arabica*	0.00075	0.75
2	F17	Bladderwrack	*Fucus vesiculosus*	0.00100	1.00
3	K06	Kelp Powder	*Laminaria Japonica*	0.00134	1.34
5	B13	Bayberry Root bark	*Morella cerifera*	0.00158	1.58
6	C8	CraneSbill Root	*Geranium maculatum*	0.00173	1.73
7	A78	Arjun	*Terminalia arjuna*	0.00183	1.83
8	P54	Ye Jiao Ten	*Polygonum multiflorum vine*	0.00288	2.88
9	P56	Mu Dan Pi	*Paeonia suffructicose root - bark*	0.00298	2.98
10	W2	Witch Hazel Root	*Hamamelis virginiana*	0.00321	3.21
11	P82	Pipsissewa	*Chimaphila umbellata*	0.00410	4.10
12	C82	Cinnamon powder	*Cinnamon powder*	0.00434	4.34
13	R19	Rose Buds and Petals Pink	*Rosa Rugosa Flower*	0.00478	4.78
14	C10	Cat Claw Bark	*Uncaria tomentosa*	0.00483	4.83
15	D10	Dryopteris Male Fern Rhizome	*Dryopteris crassirhizoma*	0.00636	6.36
16	R25	Rhodiola Root	*Rhodiola kirilowii*	0.00809	8.09
17	T30	Turkey Rhubarb	*Rheum palmatum*	0.00958	9.58
18	G26	Wintergreen	*Gaultheria procumbens*	0.01086	10.86
19	XT76	Longon Peel	*Dimocarpus longan*	0.01195	11.95
20	H12	Gloryvine Stem	*Sargentodoxa cuneata vine*	0.01260	12.60
21	M6	Meadowsweet Powder	*Filipendula ulmaria*	0.01566	15.66
22	N2	Neem Leaf	*Azadirachta indica*	0.01836	18.36
23	T25	Sang Ji Sheng	*Taxillus chinensis stem & leaf*	0.02060	20.60
24	C32	Cynomorium songaricum	*Cynomorium songaricum*	0.02150	21.50
25	P55	Chi Shao	*Paeonia Lactiflora*	0.02283	22.83
26	P83	Pygeum Bark	*Pygeum africanum*	0.02623	26.23
27	P8	Hu Zhang	*Polygonum cuspidatum rhizome*	0.02842	28.42
28	XT 8	Green Tea	*Camellia sinensis*	0.02852	28.52
29	XT91	Longon stem	*Dimocarpus longan*	0.02973	29.73
30	A1	Agrimony	*Agrimonia eupatoria*	0.03072	30.72
31	L46	Linden Leaf	*Tilia europaea*	0.03152	31.52
32	A63	Sha Ren Guang	*Amomum villosum fruit Shelled*	0.04040	40.40
33	W16	White Willow Bark	*Salix alba*	0.04108	41.08
34	W15	White Oak Bark	*Quercus alba*	0.04154	41.54
35	G40	Guarana Seed	*Paullinia cupana*	0.04253	42.53
36	S11	St Johns Wort	*St Johns Wort*	0.04408	44.08
37	L25	Lychee Pit	*Litchi chinesis seed*	0.04592	45.92
38	H4	Hawthorne Leaf and Flower	*Crataegus laevigata*	0.04790	47.90
39	P13	Bian u	*Polygonum aviculare herb*	0.05264	52.64
40	N1	Nutmeg	*Myristica fragans*	0.05811	58.11
41	S8	Saw Palmetto Berry	*Serenoa repens*	0.06608	66.08
42	M38	Maiden Hair Fern	*Adiantum capillus*	0.06847	68.47
43	T4	Truja twigs, c/s	*Thuja occidentalis Cupressaceae*	0.07676	76.76
44	P29	Wei Ling Cai	*Potentilla chinensis herb*	0.07772	77.72
